# Postural Stability in Single-Leg Quiet Stance in Highly Trained Athletes: Sex and Sport Differences

**DOI:** 10.3390/jcm11041009

**Published:** 2022-02-15

**Authors:** Nebojša Trajković, Darjan Smajla, Žiga Kozinc, Nejc Šarabon

**Affiliations:** 1 Faculty of Sport and Physical Education, University of Niš, Čarnojevićeva 10a, 18000 Nis, Serbia; nele_trajce@yahoo.com; 2 Faculty of Health Sciences, University of Primorska, Polje 42, SI-6310 Izola, Slovenia; darjan.smajla@fvz.upr.si (D.S.); ziga.kozinc@fvz.upr.si (Ž.K.); 3 Human Health Department, InnoRenew CoE, Livade 6, SI-6310 Izola, Slovenia; 4 Andrej Marušič Institute, University of Primorska, Muzejski trg 2, SI-6000 Koper, Slovenia; 5 Laboratory for Motor Control and Motor Behavior, S2P, Science to Practice, Ltd., Tehnološki Park 19, SI-1000 Ljubljana, Slovenia

**Keywords:** postural sway, balance, equilibrium, elite athletes, gender effect

## Abstract

This study aimed to determine if there is a difference in postural stability in highly trained adolescents and young adult athletes regarding sex and sport. The participants were young athletes (*n* = 464) from seven different sports. We considered the center of pressure (CoP) velocity (total, anterior–posterior (AP) and medial–lateral (ML)), CoP amplitude (AP and ML), and CoP frequency (AP and ML), as assessed by single-leg quiet stance test. Significant interactions were found between sex and sport for all CoP variables (*p* < 0.02). Additionally, a significant main effect of sport was also found in all CoP variables (*p* = 0.01). Regarding sex, significant effects were found for all CoP amplitude variables (*p* = 0.01), as well as for CoP velocity variables, except for CoP ML (*p* = 0.06). Moreover, there was no sex effect for CoP frequency AP (*p* = 0.18). The results of the current study confirm the claim that the criteria for optimal postural strategies for elite athletes likely depend on a given sport.

## 1. Introduction

Postural stability is considered a very important factor for athletes in different sports [1]. Due to its potential role in mitigating risk for injuries, postural stability has been the subject of interest of researchers. Postural stability evaluated through assessment of body sway enables quantifying the function of maintaining equilibrium during periods of standing still, locomotion, and any activities requiring a high degree of balance performance [2]. Evidence from a systematic review [3] suggests that athletes sway less than nonathletes and that highly trained elite athletes sway less than low-level athletes. The importance of good stability in some sports, (i.e., ballet, dance, gymnastics) is obvious. Previous comparisons of body sway among athletes from different sports have shown that gymnasts have better postural stability than football players, swimmers, and basketball players [4,5]. Negahban et al. [6] suggested that elite athletes may be more efficient in conditions consistent with their main experience and process of training. Accordingly, recent evidence suggests that sport-specific expertise induces alterations in sensory integration that underpins spatial referencing and postural control [7].

Novel research indicates that postural stability in athletes is not influenced by sex [8]. However, some studies noted better postural stability in females, which was suggested to be related to earlier physical and psychological maturation processes [9] and superior sensory integration [10]. Previous research has documented that female athletes have different anatomical characteristics, which could explain lower postural sway [11]. The development of proprioception and vestibular functions in females is also one factor that could interact with the improvement of the postural stability system [12]. These sex differences and inconsistency during childhood and adulthood were confirmed in several studies [13,14,15,16], indicating that girls tend to have better postural stability during childhood, while during adulthood, the situation is reversed. Therefore, improving the understanding of sport-specific patterns in postural stability and its interaction with sex is important, in order to develop better injury prevention programs and decrease injury risk.

Single-leg body sway parameters can be used for analyzing the static performance of stabilization in the condition of unilateral distribution of body weight, which is usual in sports activities. Good single-leg stabilization characteristic reflects on the smaller increase in vertical force and the shorter weight transfer in different movement tasks [17,18]. The single-leg stance test is also recommended for clinicians as a useful tool for a brief assessment of the risk of falling [19]. Therefore, evaluation of postural sway in single-leg tests presents an important stability evaluation tool. The importance of postural stability in sport and everyday life has been well recognized and confirmed. However, only a few studies investigated postural stability considering sport and sex using different tests [20,21]. Moreover, most studies were conducted on children or older adults [9,11,12,13,15,17]. Having in mind that single-leg stance measurement has more applications in clinical and sport medicine settings [22] and that most injurious falls occurred in activities that involved single-leg stance [23], it is of great importance to understand the possible sport-specific characteristics of postural stability. Accordingly, there is a widespread call to identify a postural stability measure that can best distinguish between different sports and sex in highly trained young athletes.

In light of the aforementioned evidence, we used a previously collected database containing more than 400 participants, who all performed single-leg body sway assessments with open eyes. We chose the single-leg stance test because of its similarity with the movements in sports that require balancing on a single leg and the fact that athletes must be able to maintain good postural stability before any kind of motor action, in order to act efficiently [24]. The aim of the study was to assess the postural stability during single-leg quiet stance in highly trained male and female young athletes from different sports. We expected to observe differences in center of pressure (CoP) characteristics between sex and sports in highly trained young athletes. 

## 2. Materials and Methods

### 2.1. Participants 

The participants in the present study were young athletes (*n* = 464) from 7 different sports. The sample was taken from the database of a larger project, exploring interlimb asymmetries and performance in athletes [25,26]. All sports groups that performed postural sway assessments and involved both male and female participants were considered. Details of the sample sizes for each group, along with baseline demographic data, are presented in Table 1. Exclusion criteria included lower leg injuries in the past 6 months and possible neurological or noncommunicable diseases self-reported by participants. Participants were given detailed information about the testing procedures and were required to sign a written informed consent form prior to the measurements. For minor participants, parents or guardians were also notified and signed an informed consent form on their behalf. The National Committee for Medical Ethics of the Republic of Slovenia approved the experimental protocol (Approval Number 0120-99/2018/5) and was conducted in accordance with the latest revision of the Declaration of Helsinki.

### 2.2. Procedures

Body sway was assessed in a single-leg stance position without footwear. Participants performed three 30 s repetitions with each leg in the single-leg position, with 60 s long breaks between repetitions. The experimenter began the acquisition after stabilization (1–2 s). The postural sway was analyzed on the preferred leg, which was determined as the leg that the participant would use to kick a ball. The hip of the opposite leg (i.e., non-standing leg) was in a neutral position (0°), and the thigh was parallel to the standing leg, while the knee was flexed at 90° and was not allowed to touch the standing leg. The standing leg’s knee was in the extended position but not hyperextended (locked). The hands were placed on the hips.

A piezoelectric platform (model 9260AA, Kistler, Winterthur, Switzerland) was used to acquire ground reaction force data at a sampling rate of 1000 Hz. The data were automatically filtered (low-pass Butterworth, 2nd order, 10 Hz) in the software MARS (version 4.0, Kistler, Winterthur, Switzerland). Additionally, data were automatically processed in MARS to obtain outcome variables of interest. For further analysis, an average of three replicates was used for all outcome variables. We considered mean CoP velocity (total, anterior–posterior (AP) and medial–lateral (ML)), CoP amplitude (AP and ML) and CoP frequency (AP and ML). CoP amplitude was determined as the average CoP sway in the AP or ML direction, calculated as the total length of the COP sway path in a given direction only, divided by the number of directional changes. CoP frequency was defined as the frequency of CoP oscillations, calculated as the number of peaks in the AP or ML direction (i.e., changes in the direction of CoP motion) divided by the measurement time [27].

### 2.3. Statistical Analysis

Statistical analysis was performed in SPSS (version 25.0; SPSS Inc., Chicago, IL, USA). Descriptive statistics were calculated and reported as mean ± standard deviation. The normality of the data distribution was checked with Shapiro–Wilk tests (*p* ≤ 0.121). A 2 × 7 MANCOVA was used to examine the interaction effect between sex and sport on a multivariate level. We used a 2 × 7 ANCOVA (between-subject design) to evaluate the sex and sports effects on body sway measures after controlling their effect for age (mean centered), body height (mean centered), and BMI (mean centered). The main effects of sex and sports estimated mean differences between men and women, and various sports players, respectively. The sex × sport interaction effect was employed to determine whether various sports players on average differ in body sway measures depending on the sex of sports players. For comparison of the sports included, the post hoc test was used. The effect sizes (ES) pertaining to ANOVA were expressed as partial eta squared (η2) and interpreted as small (<0.13), medium (0.13–0.26), and large (>0.26) [28].

## 3. Results

**Figure 1:**
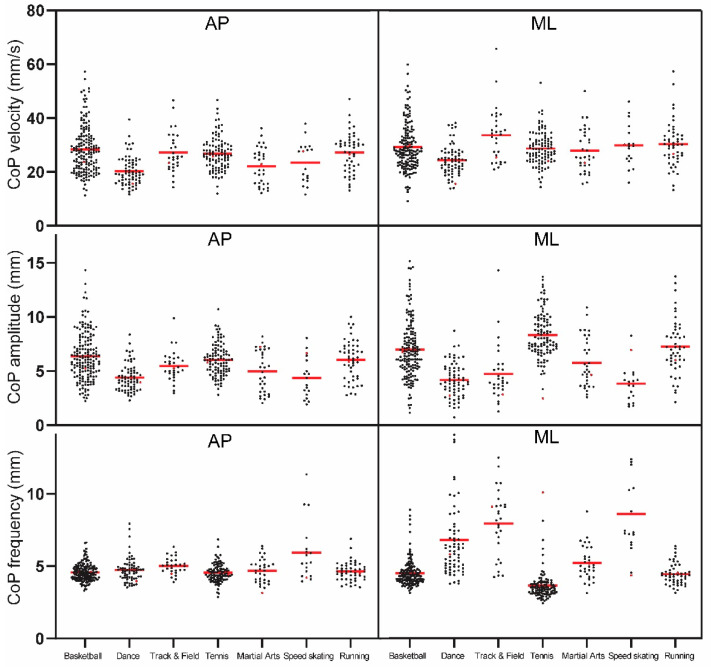
Representation of body sway data across sports (anterior–posterior direction, left panel; medial–lateral direction, right panel). Horizontal lines represent the mean of the groups). Statistical values are included in Table 2.

CoP variables, velocity, amplitude and frequency for preferred leg by sex and sports are presented in Table 2. Figure 1 represents the data for both sexes combined. A 2 × 7 MANCOVA showed that all studied variables significantly depend on sex (F = 5.936, *p* = 0.001, η2 = 0.087), sport (F = 14.614, *p* = 0.001, η2 = 0.185), and sport × sex interaction (F = 2.561, *p* = 0.001, η2 = 0.039). A 2 × 7 ANCOVA followed and showed significant interaction between sex and sport for all CoP velocity variables (*p* = 0.01) with small effect size (η2 = 0.046–0.073).There were significant main effects for sex (*p* < 0.001) and sport participation (*p* < 0.001). Concerning sex, females reported lower scores for CoP velocity than males (*p* = 0.01; η2 = 0.027–0.079, small ES), except for CoP velocity ML, where no significant effect of sex was found (*p* = 0.06; η2 = 0.08, small ES). Similarly, there was a significant effect of sports (*p* = 0.01), with small ES ranging from η2 = 0.062 to η2 = 0.093.

There were significant sex × sport interactions found for CoP frequency (CoP AP F = 4.51; *p* = 0.01; CoP ML F = 2.45; *p* = 0.02). Moreover, there was no main effect of sex for CoP AP frequency (*p* > 0.05). Regarding sport modality, there was a significant main effect for CoP AP and ML frequency (*p* = 0.01; η2 = 0.143–0.400).

## 4. Discussion

This study aimed to analyze characteristics of single-leg quiet stance body sway in a highly trained athletic population and to explore the effects of sex and sport. The major findings in the current study point to sport-specific characteristics regarding postural stability in single-leg stance. Results for CoP velocity and amplitude clearly show that dancers are better able to maintain a stable single-leg stance than athletes from other sports. Moreover, the results of the present study show that, when comparing highly trained athletes of both sexes, there were differences in almost all CoP variables. Male athletes presented higher values of CoP velocity and amplitude but also for CoP frequency during single-leg stance.

The single-leg upright stance represents a challenging part of human locomotion because, compared with bipedal stance, it requires keeping the center of body mass within the smaller area of the support [13], which leads to more corrective movements by the postural control system in order to maintain balance [29]. Female athletes in the current study reported lower scores (*p* = 0.01) for CoP velocity (η2 = 0.030–0.079) and amplitude (η2 = 0.055–0.082), compared with male athletes. Studies investigating postural control between sexes showed inconsistent results depending on the age group. Boys aged nine years showed significantly poorer single-leg postural stability than the girls of the same age [13], and similar was for participants in adolescent age [14]. On the contrary, female young adults seem to have lower postural stability, as shown by higher CoP velocities, compared with males [16]. The differences remain through adulthood as well in older adults—women tend to be less stable than men during single stance [15]. The main predictor that could influence this inconsistency in the results is the visual system, which was found to be the primary sensory system involved in maintaining postural stability in a broad range of age groups [30]. One more factor that could change the variability in postural stability is physical activity. This was confirmed in a study conducted on healthy young adults, in which no differences between sexes were detected when participants were physically active [31]. Female athletes in the current study showed better postural stability, compared with males with the difference being most pronounced for CoP velocity and amplitude, while the difference in CoP frequency was noted in the medial–lateral direction only (*p* < 0.05). Reduced CoP sway area and velocity in females of similar age were also noted in the recent study [21]. Possible reasons for better postural stability in this age are the maturation process [32] lower body weight [13], as well as better proprioception and control due to smaller absolute muscle mass and strength [33]. Additionally, it was stated that sex differences exist in children and adolescents due to the significantly lower body height in girls [13]. However, the participants in the abovementioned study were untrained children that were younger than participants in the current study, which may account for the discrepancies in some studies. Therefore, reasons for better postural control in younger age, as well as the reasons for the decline in later ages, should be investigated further.

It was stated that the postural balance of elite athletes should be always monitored, due to the establishment of sport-specific imbalances that could affect their performance [34]. The results of the current study suggest that dancers have better postural stability during single-leg stance than athletes from other sports, in all measured CoP characteristics. The differences are probably the result of adaptive balance strategies used by dancers in training, in which both abilities, cognitive and physical, are coordinated [35]. Additionally, their continuous training that uses balance control could minimize the effect of external perturbations [36] and thus improve postural control. However, the increase in body sway in the absence of vision in ballet dancers was previously reported by Bruyneel et al. [37]. Matsuda et al. [20] showed that soccer players make greater use of the somatosensory system during single-leg stance, compared with basketball players, swimmers, and nonathletes. However, highly trained female volleyball players showed higher CoP fractal dimensions, compared with controls, which is probably due to the adoption of certain habits. [38]. According to the authors, these high values show evidence for flexible and variable strategies of maintaining balance by highly trained athletes. This was confirmed in the current study among young highly trained athletes in that dancers were better able to maintain a stable, single-leg stance, compared with athletes from other sports. Only martial arts showed similar results to those of dance for CoP velocity and amplitude but also higher. The mechanism behind the best postural stability in dancers may be associated with the development of a motor skill for voluntary stabilization of important muscle groups, as well as better sensorimotor solutions for posture control [39]. The importance of dance exercise in maintaining good postural stability was well documented in adolescent females [40].

Some limitations of the present study need to be acknowledged. Across sports, the sample sizes varied considerably and were relatively small for some of the sports. Moreover, the samples were not sex balanced (e.g., dancers. Despite taking sexes into account as a factor in analyses, some main effects could still be driven by a larger representation of one sex in the sample (e.g., females in dancing). However, having in mind that, in some sports, it is hard to find highly trained athletes on an elite level, and since we included a considerable number of different sports, it was of great importance to conduct and analyze body sway because of important clinical implications in young athletes. Moreover, we did not include a healthy control group, which could have strengthened our interpretations. One more limitation is the fact that measures were assessed only during static conditions. Most of the selected sports rely more on dynamic conditions demands than static postures. Therefore, future studies should use both dynamic and static postural tests, in order to provide an overall assessment of balance in different sports. Nevertheless, the greatest strength of this study is encompassing a large number of sports and highly trained athletes compared at this age. 

## 5. Conclusions

According to our findings, postural stability in highly trained adolescents and young adult athletes was influenced by sex and sport. Female athletes showed better postural stability than male athletes. The athletes engaged in dance showed the highest postural control, compared with other sports. The results of the current study confirm the claim that the criteria for optimal postural strategies for elite athletes likely depend on a given sport. This is of great importance in providing additional information about postural control abilities in highly trained athletes from different sports.

## Figures and Tables

**Table 1:** Basic participant data.

	*n*	Age (years)	Body Height (cm)	Body Mass (kg)	Weekly Training	Years of Training
Basketball—M	107	17.4 (2.2)	189.3 (8.2)	81.4 (12.9)	6.4 (1.9)	6.9 (2.4)
Basketball—F	58	16.7 (1.6)	175.2 (5.6)	70.2 (11.2)	5.5 (1.3)	6.4 (2.5)
Dance—M	23	24.2 (5.9)	179.0 (4.9)	71.7 (6.6)	5.9 (2.2)	12.0 (4.4)
Dance—F	54	22.3 (7.0)	166.9 (5.3)	55.3 (6.1)	6.6 (2.6)	9.9 (4.0)
Track and Field—M	21	17.8 (2.6)	180.5 (5.8)	73.8 (7.9)	5.4 (1.6)	6.5 (3.1)
Track and Field—M	8	17.7 (3.0)	167.2 (3.7)	60.3 (5.8)	5.4 (1.1)	6.3 (2.2)
Running—M	31	29.2 (8.8)	181.2 (5.6)	77.2 (6.8)	5.2 (2.5)	11.0 (8.8)
Running—F	18	36.9 (10.9)	166.0 (8.1)	60.9 (7.6)	4.0 (1.7)	7.7 (4.5)
Tennis—M	68	17.2 (10.4)	175.0 (11.1)	65.2 (12.1)	6.1 (2.8)	8.9 (3.6)
Tennis—F	42	15.9 (3.0)	168.5 (8.4)	60.0 (9.9)	6.3 (3.2)	8.2 (3.9)
Martial arts—M	18	19.9 (3.1)	180.3 (6.0)	75.5 (8.9)	5.6 (1.3)	7.7 (2.5)
Martial arts—F	17	19.7 (3.4)	169.1 (6.6)	60.1 (5.1)	5.1 (1.4)	7.7 (2.8)
Speed skating—M	12	16.8 (5.1)	169.5 (15.5)	61.3 (16.5)	5.3 (1.9)	6.9 (3.4)
Speed skating—M	7	16.9 (3.4)	161.1 (8.4)	57.3 (10.9)	4.9 (2.0)	6.0 (3.9)

M—male; F—female.

**Table 2:** Comparison of CoP velocity, amplitude, and frequency according to sex and sport; values are mean ± SD. Statistically significant effect are in bold.

		Basketball	Dance	Track and Field	Tennis	Martial Arts	Speed Skating	Running	Sport × Sex F; *p* Value	Sport F; *p* Value	Sex F; *p* Value
CoP velocity (mm/s)											
Total	male	49.8 ± 12.8 b	37.2 ± 10.6 ac	49.1 ± 13.9 b 45.2 ± 10.2 be	45.1 ± 8.8	47.1 ± 8.9	44.1 ± 9.7 38.5 ± 13.4	44.4 ± 9.5 45.6 ± 14.8 be	**5.01; 0.01**	**5.58; 0.01**	**12.31; 0.01**
	female	36.3 ± 8.0	34.4 ± 6.3 g		41.3 ± 8.8 b	31.6 ± 6.8 g					
AP	male	31.5 ± 8.7 be 22.1 ± 19.9	21.2 ± 6.9 acd 19.9 ± 4.5 dg	28.9 ± 7.9 b 22.9 ± 4.3 b	27.7 ± 6.1 ab 25.2 ± 6.0 be	27.4 ± 4.4 16.9 ± 4.1 dg	26.2 ± 7.1	26.9 ± 6.1 27.6 ± 8.9 be	**5.85; 0.01**	**7.67; 0.01**	**24.44; 0.01**
	female						19.2 ± 6.7				
ML	male	32.2 ± 8.6 24.2 ± 5.8 c	26.1 ± 7.1 c	33.6 ± 10.5 b 34.1 ± 9.4 abe	29.7 ± 6.1 27.4 ± 5.7	32.8 ± 7.5	30.2 ± 5.9 29.6 ± 10.7	30.1 ± 6.9 31.0 ± 10.1	**3.94; 0.01**	**5.62; 0.01**	3.68; 0.06
	female		24.0 ± 4.6 c			23.1 ± 5.2 c					
CoP amplitude (mm)											
AP	male	7.1 ± 2.1 bcf	4.5 ± 1.2 adeg	5.6 ± 1.5 a	6.2 ± 1.5 b 5.6 ± 1.3 bef	6.3 ± 1.2 b 3.6 ± 1.3 d	5.1 ± 1.8 a 3.0 ± 0.8 d	6.3 ± 1.7 b 5.5 ± 1.9	**5.97; 0.01**	**8.17; 0.01**	**21.43; 0.01**
	female	4.8 ± 1.3	4.3 ± 1.3 d	5.0 ± 1.1							
ML	male	7.9 ± 2.6 bcf	4.7 ± 1.6 adeg	4.6 ± 2.8 adeg	8.4 ± 2.1 bcf 7.9 ± 2.1 abcefg	7.3 ± 1.9 bcf 4.1 ± 1.1 d	4.3 ± 1.8 ade 2.9 ± 1.2 adg	7.5 ± 1.6 bc 6.7 ± 2.8 bdf	**3.86; 0.01**	**26.37; 0.01**	**7.01; 0.01**
	female	5.2 ± 1.7 bdf	3.8 ± 1.4 adg	4.7 ± 2.0 d							
CoP Frequency (Hz)											
AP	male	4.5± 0.5 cf	4.7± 0.9	5.1± 0.4 ad	4.5± 0.6 cf 4.4 ± 0.5 fg	4.3± 0.6 f 4.9 ± 0.9 f	5.4 ± 1.5 ad	4.3 ± 0.4 f	**4.51; 0.01**	**10.36; 0.01**	1.82; 0.18
	female	4.7± 0.6 f	4.7 ± 0.7 f	4.6 ± 0.6 f			6.6 ± 2.6 abcdeg	5.1 ± 0.6 df			
ML	male	4.2 ± 0.6 bcf	5.8 ± 1.5 acdg	8.0 ± 2.3 abdeg	3.7 ± 1.1 bcf 3.5 ± 0.9 abcef	4.6 ± 0.7 cf 5.8 ± 1.2 df	7.7 ± 2.5 adeg 10.2 ± 3.4 abdeg	4.2± 0.6 bcf 4.8 ± 0.7 bcg	**2.45; 0.02**	**46.42; 0.01**	**8.22; 0.01**
	female	5.0 ± 1.1 bcdf	7.2 ± 3.5 adfg	7.8 ± 2.4 adg							

a—significant differences from basketball; b—significant differences from dance; c—significant differences from track and field; d—significant differences from tennis; e—significant differences from martial arts; f—significant differences from speed skating; g—significant differences from running; AP—anterior–posterior; ML—medial–lateral; statistically significant sex × sport interactions were found for CoP AP amplitude (*p* = 0.01), as well as in CoP ML amplitude (*p* = 0.01), although the effect sizes were all small (η2 = 0.023–0.078). There was a significant main effect of sex and sport for both CoP AP and CoP ML amplitude (*p* = 0.01). Effect size ranged from small (η2 = 0.087) to large (η2 = 0.263) regarding sport, and from 0.022 to 0.082 regarding sex.

## Data Availability

The data presented in this study are available on request from the corresponding author.
